# Late Effects After Haematopoietic Stem Cell Transplantation in ALL, Long-Term Follow-Up and Transition: A Step Into Adult Life

**DOI:** 10.3389/fped.2021.773895

**Published:** 2021-11-24

**Authors:** Tamara Diesch-Furlanetto, Melissa Gabriel, Olga Zajac-Spychala, Alessandro Cattoni, Bianca A. W. Hoeben, Adriana Balduzzi

**Affiliations:** ^1^Division of Pediatric Oncology/Hematology, University Children's Hospital Basel (UKB), University of Basel, Basel, Switzerland; ^2^Cancer Centre for Children, The Children's Hospital at Westmead, Westmead, NSW, Australia; ^3^Department of Pediatric Oncology, Hematology and Transplantology, University of Medical Sciences, Poznań, Poland; ^4^Clinica Pediatrica, University degli Studi di Milano-Bicocca, Fondazione Monza e Brianza per il Bambino e la sua Mamma (MBBM), San Gerardo Hospital, Monza, Italy; ^5^Department of Radiation Oncology, University Medical Center Utrecht, Utrecht, Netherlands; ^6^Princess Máxima Center for Pediatric Oncology, Utrecht, Netherlands

**Keywords:** haematopoietic stem cell transplantation, long-term survivors, quality of life, paediatric, adolescence, late effects, ALL

## Abstract

Haematopoietic stem cell transplant (HSCT) can be a curative treatment for children and adolescents with very-high-risk acute lymphoblastic leukaemia (ALL). Improvements in supportive care and transplant techniques have led to increasing numbers of long-term survivors worldwide. However, conditioning regimens as well as transplant-related complications are associated with severe sequelae, impacting patients' quality of life. It is widely recognised that paediatric HSCT survivors must have timely access to life-long care and surveillance in order to prevent, ameliorate and manage all possible adverse late effects of HSCT. This is fundamentally important because it can both prevent ill health and optimise the quality and experience of survival following HSCT. Furthermore, it reduces the impact of preventable chronic illness on already under-resourced health services. In addition to late effects, survivors of paediatric ALL also have to deal with unique challenges associated with transition to adult services. In this review, we: (1) provide an overview of the potential late effects following HSCT for ALL in childhood and adolescence; (2) focus on the unique challenges of transition from paediatric care to adult services; and (3) provide a framework for long-term surveillance and medical care for survivors of paediatric ALL who have undergone HSCT.

## Introduction

Haematopoietic stem cell transplant (HSCT) is a curative option for children and adolescents with haematological malignancies, especially patients with high-risk, relapsed or refractory disease (1–3). Given improvements in treatment modalities and supportive care, the number of long-term survivors of paediatric HSCT is growing continuously (4–6). However, pre-transplant treatment exposure, transplant conditioning regimens and transplant-related complications are associated with a wide range of adverse late effects, resulting in a shorter life expectancy compared with sex- and age-matched healthy subjects and patients treated with conventional chemotherapy alone (4, 7–12). Every system and organ can be affected by the long-term sequalae of HSCT, resulting in higher morbidity and reduced overall quality of life (QoL) compared with patients with ALL treated with chemotherapy only (13–15). One year after HSCT, ≥30% of all transplanted patients have developed at least one severe late effect (12). A major risk factor for development of severe late effects is young age at the time of treatment, with children <3 years being especially at risk due to their vulnerable developing organs (16). Table 1 shows the reported frequency of different long-term sequalae.

**Table 1:** Reported frequency of long-term sequalae in ALL survivors treated with HSCT during childhood.

**Organ/organ system**	**Reported frequency (%)**	**Long-term sequelae**	**Risk factors**	**Long-term follow-up TBI-based**
Ocular system	4–76% 35–77% 0–10%	- Cataract - Sicca syndrome - Microvascular retinopathy	TBI, long-term corticoid treatment, GvHD, busulfan treatment	- Ophthalmological examination^*^^∙^
Cardiovascular system	6% 39% 10–50% 22%	- Cardiovascular dysfunction - Metabolic syndrome - Arterial hypertension - Coronary artery, cerebrovascular and peripheral arterial disease	TBI, anthracycline, hemosiderosis	- Endurance training - Dietary measures - Weight reduction - Antihypertensive treatment
	2–10%	- Cardiac arrhythmias/heart failure	
Lungs	11–67%	- Bronchiolitis obliterans	TBI, busulfan treatment, cGvHD	- Lung function^*^^∙^
Renal system	20% 2–21%	- Chronic renal insufficiency - Thrombotic microangiopathy	TBI, nephrotoxic treatment	- Urinary status
Liver	30–75%	- Iron overload	Cumulative number of transfusions, viral infections, cGvHD, drug toxicity	- Liver function test - Phlebotomy in hemosiderosis
Bone	4–40% 5–21%	- Osteonecrosis - Low bone mineral density	TBI, hypogonadism, physical inactivity, long-term corticosteroids, cGvHD	- Physiotherapy - Calcium and vitamin D supplementation
Endocrine system	20–40% 5–17% 44–100% 15–55% 75–100% 5% Diabetes mellitus 50% insulin resistance 30%	- Growth retardation - Thyroid disorders - Ovarian insufficiency - Testosterone deficiency - Oligo-azoospermia - Hyperinsulinism, impaired glucose tolerance, diabetes - Hypercholesterolemia/ hyperlipidemia	TBI, long-term corticoid treatment, cGVHD, calcineurin and mTOR inhibitors Parenteral nutrition	- Ultrasound of thyroid^*^ - Hormone replacement therapy - Dietary measures - Statin in case of high cholesterol
Eyes	4–76% 35–77% 0–10%	- Cataract - Sicca syndrome - Microvascular retinopathy	TBI, long-term corticoid treatment, GvHD, busulfan treatment	- Ophthalmological examination^*^^∙^
Kidney	20% 2–21%	- Chronic renal insufficiency - Thrombotic microangiopathy	TBI, nephrotoxic treatment	- Urinary status

* *TBI-based regimen*.

∙ *Busulfan*.

Most conditioning regimens used in children are myeloablative and contain busulfan, cyclophosphamide, etoposide or total body irradiation (TBI). Patients >4 years old receiving TBI-based conditioning have a significantly lower risk of ALL relapse and higher overall survival than patients receiving chemotherapy alone as conditioning regimen, as demonstrated in the randomised For Reducing Radiation at Majority Age (FORUM) Trial (17). However, patients receiving TBI may have to deal with a higher risk of pubertal impairment, growth retardation, cataracts, secondary malignancies and thyroid dysfunction compared with patients undergoing TBI-free conditioning (18).

A history of graft-vs.-host disease (GvHD) increases the risk severe life-threatening conditions 4.7-fold, and is therefore associated with higher morbidity and mortality. Overall, the cumulative incidence of developing a chronic health condition 10 years after HSCT for patients with a haematological malignancy or severe aplastic anaemia transplanted between 1974 and 1998 was about 59% [95% confidence interval (CI) 56–62%] (19).

Due to the high burden of long-term sequelae, survivors of HSCT performed during childhood and adolescence need regular, life-long follow-up. Goals of follow-up include the early detection of potential long-term effects and the education of survivors and their families to promote a healthy lifestyle. It is also important that providers of paediatric and adult healthcare are trained to facilitate and optimise the transition of patients into adult care (20).

## Organ-Specific Late Effects of HSCT for Childhood and Adolescent All

### Bone

Bone morbidity is frequently occurs after HSCT in ALL, with reported incidences ranging between 20 and 60% for reduced bone mass, spanning from low mineral density to osteoporosis, and 4–44% for osteonecrosis (ON) (13, 21).

Besides the skeleton, muscles as well are deeply affected by treatment toxicity, especially by steroid, chemotherapy-induced peripheral neuropathyas well as by bed rest and reduced physical activities during treatment.

Associated with these sensory and motor symptoms is a compromised ability to move that leads to functional impairment in transplanted patients (22). Chronic GvHD may target the muscular mass by direct inflammation of the tissue (23).

#### Low Bone Mineral Density

A bone mineral density (BMD) Z score below −2 has been recorded in about 6–21% of patients 5 years after childhood HSCT for either malignant or non-malignant disorders (24–30). Nevertheless, this incidence is remarkably higher in patients with additional risk factors, such as chronic GvHD. In a study held among patients with a longstanding history of chronic GvHD transplanted for either malignant or non-malignant disorders, Buxbaum and colleagues reported BMD Z score < −2 in 73% of patients after a median follow up of 3.5 years (25).

The aetiology of low BMD is multifactorial, with prolonged exposure to corticosteroids, immobility, TBI, hormonal deficiencies (hypogonadism, hypothyroidism, GHD), inadequate vitamin D and calcium intake and GvHD having synergic detrimental effects and resulting in overactive osteoclastic bone resorption and underactive bone osteoblastic formation (31).

Dual energy X-ray absorptiometry (DEXA) is pivotal to assess BMD, although the best timing for performing this test has not been systematically clarified. Recommendations based on expert opinion suggest performing the first DEXA scan 1 year after HSCT, with subsequent follow-up tailored based on the baseline findings and patient-related risk factors (32).

The dietary calcium and vitamin D intake of every ALL survivor post HSCT should be assessed in order to identify those patients who would benefit from supplements. In addition, given the detrimental effects of low sexual hormones on BMD, hormonal replacement therapy should be commenced as soon as a diagnosis of hypogonadism is made.

There is lack of high-quality data about the predictive role of low BMD in childhood on the incidence of fractures in adulthood and uncertainty about the risk–benefit balance of pharmacological treatments (bisphosphonates) in childhood. Therefore, the decision of when to intervene with such treatments to prevent or treat bone disease in children after HSCT who have not experienced pathological fracture should be made in consultation with a paediatric endocrinologist; no treatment guidelines were available at the time of writing (26, 33, 34).

#### Osteonecrosis

Osteonecrosis (ON) is a well-known sequela in paediatric ALL. Incidence is age-dependent and ranges from 4 to 44% in transplanted patients, with cumulative steroid dose being significantly associated with the risk of ON (35). The pathogenesis of osteonecrosis in patients with ALL is not completely understood, multiple factors are responsible Main cause in childhood ALL are glucocorticoid inducing a hypercoagulable state. Microthrombi and lipid emboli associated with hyperlipidemia cause intravascular obliteration, whereas lipocyte proliferation and lipid accumulation in osteoblasts and osteocytes cause extraluminal obliteration, both triggering and worsened by intravascular coagulation. ON pathogenesis in ALL includes the temporary or permanent disruption of the blood supply to the bone, glucocorticoid-induced arteriopathy and direct adverse effects of the antileukemic drugs on bone remodelling (21). Furthermore, there is mounting evidence that post-HCT cGvHD, besides increasing the steroid cumulative dose, could play an independent pathogenetic role in the development of ON, possibly mediated by microangiopathy (36).

Most frequent symptoms at diagnosis are bone pain, decreased mobility in a joint, and limping. Incidence and risk factors differ substantially among studies, even after chemotherapy alone, from 2%, as reported in the AIEOP-ALL 95, to 25% in the CoALL-07-03 (37–40). Such a variability may depend on different frontline and second line treatment strategies, including age eligibility and cumulative dose of steroids and other drugs, mainly asparaginase, besides ON diagnostic approaches, based on the level of alertness among physicians, experience of radiologists and orthopaedics (21). ON is often misdiagnosed in children and adolescents, in whom symptoms may vary from stiffness to pain often attributed to the ongoing chemotherapy courses. The true prevalence of ON, however, is unknown, as it can only be determined by prospective MRI screening the most sensitive method of ON detection (38).

There is a consensus on the effect of age, with adolescents and young adults being at highest risk of ON, compared with younger children, whereas the impact of gender and immunophenotype on the risk of ON is still controversial. Risk stratum and associated treatment strategy are likely to play a major role.

The impact of HSCT in increasing the incidence and worsening the severity of ON can be hard to assess, as lesions are often present prior to HCT, as shown by MRI performed as pre-HCT screening often detecting pre-existing ON in patients with relatively mild symptoms (21, 35, 41, 42).

Kuhlen assessed the risk of ON in a cohort of 557 evaluable patients transplanted within the ALL SCT 2003 BFM trial. The 5-year cumulative incidence of symptomatic ON was 9%, diagnosed at a median of 1 year after HCT (range 1–126). Age at HSCT was a risk factor, with adolescents having a 3.73-fold (10–15 years; *P* = 0.009) to 5.46-fold (>15 years; *P* = 0.001) higher of being diagnosed with a symptomatic ON, compared with children. Patients with a history of ON prior to HSCT were at increased risk (HR 5.45, *P* = 0.001), with a cumulative incidence of ON of 45% (SD 14%) compared with 9% (SD 2%) in those without ON prior to HSCT (*P* < 0.001). Furthermore, the presence of chronic GVHD was associated with a 2.7-fold higher risk (*P* 0.015) for the development of ON. Neither gender, remission phase, donor type, stem cell source, type of conditioning regimen or aGvHD grade 2–4 were significant risk factors (21, 43).

Most patients have multiple lesions at diagnosis (42% in the ALL SCT Trial), mainly in the lower limbs, namely knees (66%), hips (55%), and feet (50%), but also in the shoulders (22%) (21). Lesions affecting joint surfaces in the lower limbs experienced the worst evolution (42).

ON management is still controversial, as, beyond pain management and physical therapy, most interventions lack clinical evidences. Treatment of prolonged hypertriglyceridemia/hypercholesterolemia, e.g., dietary measure, omega3-fatty acids throughout chemotherapy and during the post-HSCT course may help in reducing ON risk. Crutches are often recommended in order to avoid weight-bearing in lower limb ON, but their use is controversial, as the absence of weight may weaken the bone structure and crutches *per se* may worsen misdiagnosed ON lesions in the upper limbs.

There is no consensus for type and timing of surgery, which include conservative procedures, as core decompression, with the aim to reduce intraosseous pressure and promoting healing processes, sometimes in combination with autologous or mesenchymal stem cells, to invasive procedures, as arthroplasty and joint replacement.

### Cardiovascular System

HSCT recipients surviving long term have a higher risk of cardiovascular (CV) dysfunction than the general population. The incidence of late CV complications in HSCT recipients is up to 6%, with the risk of premature CV-related death increased 2.3-fold compared with the healthy individuals (44–46). The aetiology of CV-related deaths in cancer survivors, including those after HSCT, is multifactorial, including anthracycline-associated congestive heart failure, radiation-induced cardiac toxicity or other causes that may be disease or treatment related in nature (45, 47, 48). Recognising the heterogeneity of risk factors and CV complications, here we focus on the following overarching topics: CV risk factors (mainly metabolic syndrome), arterial disease, and cardiac dysfunction.

#### Cardiovascular Risk Factors

Metabolic syndrome is a constellation of central obesity, insulin resistance, glucose intolerance, dyslipidaemia, and hypertension, and can be found in 39% of ALL survivors following HSCT vs. 8% of patients with leukaemia treated with conventional chemotherapy only (45). Risk for development of atherosclerotic CV disease is substantially elevated following HSCT when compared to the general population (49). While dyslipidaemia and other metabolic abnormalities are common after HSCT, often as a side effect of immunosuppressive treatments such as calcineurin inhibitors for GvHD, some of these abnormalities may resolve after cessation of immunosuppressive treatment. Nevertheless, laboratory data from paediatric allogeneic HSCT recipients 1 year post transplant suggests that those with higher total cholesterol and triglyceride serum concentrations may be more likely to experience a subsequent serious CV event (46).

Hypertension is another complication observed in both adult and paediatric HSCT recipients; rates ranging from 10 to 50% have been reported, with the variation due to differences in population composition, follow-up length and assessment method. Risk factors for hypertension among HSCT recipients include increasing age, the presence of obesity and other CV risk factors. While immunosuppressive medications used to treat GvHD often are associated with acute hypertension, evidence for acute or chronic GvHD as a risk factor for persistent hypertension once survivors are off immunosuppression is mixed (50). Similarly, while TBI, kidney injury and male sex have been postulated to be potential risk factors for hypertension, they have not been consistently found to be independent risk factors in clinical studies. Nevertheless, since it is difficult to predict whether hypertension, dyslipidaemia, or diabetes developing soon after HSCT will later spontaneously resolve, tighter control of these CV risk factors soon after they manifest may be more appropriate than watchful waiting. Certainly, ALL survivors who received HSCT and who have pre-existing CV risk conditions should continue to be monitored closely and treated for these conditions (46, 50).

#### Arterial Diseases

Arterial diseases including coronary artery disease, cerebrovascular disease and peripheral artery disease are diagnosed in up to 22% of HSCT recipients at 20 years after transplant and have emerged as the most important cause of CV-related mortality in long-term survivors from paediatric cancer (51). Atherosclerosis is a complex process involving inflammation and cellular proliferation in arterial walls. The development and progression of atherosclerosis is mediated by a variety of growth factors, cytokines, thrombotic factors and vasoactive substances. Additional modifying factors that have been implicated include: endothelial injury induced by radio-chemotherapy (radiation, alkylating agents, platinum agents, and high-dose cyclophosphamide conditioning), GvHD, immunosuppressive agents and other endocrine disorders (e.g., gonadal dysfunction). Host genetic polymorphisms may be involved in modulation of arterial disease risk after HSCT; however, to date no specific genetic variant has been described (44, 52). As screening for subclinical arterial disease is limited due to the lack of standardised and reproducible methods, prevention recommendations applicable to the general population (including lifestyle modifications and/or prophylactic pharmacotherapy) are the only known ways to reduce the risk of arterial disease in HSCT recipients.

#### Cardiac Dysfunction

The most important cardiac dysfunctions observed in ALL survivors after HSCT are heart failure and cardiac arrhythmias, being observed in 2–10% of all survivors (44). Predisposing factors for early heart failure in ALL patients who have undergone HSCT include reduced pre-HSCT ejection fraction, conditioning with high-dose cyclophosphamide and TBI. The risk of late-occurring heart failure is primarily attributable to pre-HSCT anthracycline exposure, in a dose response manner (7). Moreover, the risk increases significantly among those who also have conventional CV risk factors such as hypertension and/or diabetes. Early screening by echocardiography for asymptomatic disease may provide opportunities for implementation of interventions to reduce the risk of clinically overt disease; e.g., ACE inhibitors for asymptomatic left ventricular dysfunction. Thus, according to COG recommendation routine assessment of cardiac function (systolic and diastolic) using two-dimensional echocardiography should be performed at intervals ranging from yearly to every 5 years, depending on their exposure doses and age at exposure. Cardiac arrhythmias can have serious health implications in HSCT recipients but often cause no symptoms. The focus of long-term care is to identify and treat arrhythmias that may eventually result in symptomatic disease and to treat other CV complications such as stroke, haemodynamic collapse. Existing post-HSCT care guidelines do not recommend routine screening by electrocardiogram or Holter monitoring in patients without symptoms or a concerning family history (7, 44, 53), although consideration for screening in all ALL survivors who have had HSCT is suggested.

### Endocrine System

Endocrinopathies, reported in nearly 60% of patients transplanted before the age of 10 years, represent the most frequent sequelae after paediatric HSCT (54). The endocrine late effects experienced by transplanted ALL survivors include poor growth, thyroid disorders, gonadal insufficiency, impaired glucose homeostasis, and reduced bone mineral density (55).

In ALL patients, the overall odds of developing an endocrinopathy mostly depends on the treatment intensity delivered at frontline and as a part of the conditioning regimen, with cranial radiotherapy (56, 57) and a higher cumulative dose of alkylators and TBI (58) being the most detrimental determinants. Single-fraction TBI has been demonstrated to expose survivors to a lifelong and remarkably higher risk of endocrinopathy when compared with fractionated protocols (59). Although TBI is currently delivered in multiple fractions by the vast majority of radiotherapy centres, data about historical conditioning is pivotal to assess the risk in the large number of long-term survivors followed-up in late effects clinics. Of note, patients who received HSCT after TBI-free conditioning are also at risk of developing multiple endocrinopathies (60). Furthermore, host-related variables (i.e., age at HSCT), steroid cumulative dose and chronic GvHD may have additional detrimental effects on the endocrine system (61).

#### Linear Growth

Impaired growth and short stature at final height attainment are the result of a combination of hormonal and non-hormonal detrimental factors among transplanted ALL patients. These factors include decreased nutritional intake, psychosocial issues, high-dose corticosteroids, hypogonadism and hypothyroidism (55). Radiation-induced growth hormone deficiency (GHD) commonly represents the only hypothalamic-pituitary deficiency experienced after low doses of radiotherapy (12–24 Gy) delivered with prophylactic cranial radiotherapy or TBI (62). GHD has been reported in 20–40% of HSCT recipients conditioned with TBI for haematological malignancies (63–65), with most of this variability being a result of discrepancies in the diagnostic criteria and on the radiation dose delivered. Younger age at radiation involving the hypothalamic-pituitary area (66, 67) and TBI provided as a single fraction are negative prognostic factors (59). As recommended by the Endocrine Society, recombinant human growth hormone (rhGH) can be administered to patients with demonstrated GHD and a stable oncological remission for ≥12 months after the discontinuation of antineoplastic treatments and after a thorough discussion of risks and benefits with the caregiver (68). Nevertheless, TBI, especially after cumulative total doses of more than the equivalent of 15 Gy in 2-Gy fractions, has been demonstrated to affect growth in a GH-independent manner through radiation-induced damage involving the growth plates (66, 69–71). Younger children are more affected and single-dose TBI causes a greater decrease in final height than fractionated TBI. As a result, short stature occurs after TBI also in patients without GHD. In addition, rhGH fails to restore growth potential in individuals with GHD who had undergone TBI, with over 60% of treated patients failing to reach their mid-parental stature at final height (72–75). Nevertheless, a measurable beneficial effect of rhGH on growth and adult height in GHD patients has been demonstrated also after TBI (76).

Finally, tyrosine kinase inhibitors (which are administered to Philadelphia chromosome-positive ALL patients), have been widely described as disruptors of the GH–insulin-like growth factor I (IGF-I) axis, potentially leading to growth impairment. Nevertheless, the effect tends to be remarkably less evident than in patients treated chronically, as in chronic myeloid leukaemia (77).

#### Thyroid Disorders

Hypothyroidism, often subclinical, has been widely reported after TBI as well as following busulfan- and cyclophosphamide-based conditioning therapy (78–83). In a study published in 1997 regarding 270 young adult patients transplanted for haematological malignancies, Al-Fiar et al. identified raised thyroid-stimulating hormone levels within 2 years of allogeneic HSCT in 11% of patients after chemo-conditioning vs. 16.7% after 12 Gy fractionated TBI (84). In the same year, Toubert et al. reported hypothyroidism during a 14-month follow-up in 14% of a cohort of 77 patients transplanted in childhood or young adulthood for either malignant or non-malignant haematological disorders after conditioning regimens that did not include TBI (83). Nevertheless, it has been suggested that these finding may for a large part be transient, especially after chemotherapy-only conditioning.

Radiation involving the neck provides a direct detrimental effect to the thyroid gland (85). However, it is also associated with an increased incidence of autoimmune thyroid disorders, probably because autoantigens may be released from damaged thyroid glands and recognised by the immune system (86, 87). Prolonged immunosuppression and GvHD seem to play a contributory role (88).

HSCT is an independent risk factor for the development of thyroid nodules and malignancies. In a large French study published in 2016 following 502 transplanted childhood ALL survivors, the incidence of thyroid malignancy was 5.2%, with a cumulative incidence of 9.6% at 20 years (89). Although the detrimental role of TBI, especially when delivered as an unfractionated dose in patients younger than 10 years, has been known for decades (81, 88), an increasing body of knowledge has shed light on the harmful role that is also played by alkylating agents in this setting (79, 89). As the median average time elapsed between diagnosis of ALL and thyroid cancer is as long as 16 years, lifelong monitoring for patients is mandatory (79, 89).

#### Ovarian Insufficiency

Impaired gonadal function is the most frequent endocrine sequelae among transplanted ALL female survivors, as both alkylating agents and radiotherapy halt follicle maturation and cause a rapidly progressing depletion of ovarian follicles (3, 90). In pre- or peri-pubertal girls, the occurrence of pubertal delay or arrest prompting the need for pharmacological hormonal induction to achieve menarche depends on the conditioning regimen received, with an incidence of 16% after cyclophosphamide alone, 72% after busulfan plus cyclophosphamide, 71% after 10 Gy of single fraction TBI and 57% after 12–15.75 Gy of fractionated TBI (80, 91). In this setting, the administration of progressively increasing doses of oestrogen initially (80) provides the patient with secondary sexual features, while the subsequent cyclical addition of progestins prompts the occurrence of withdrawal bleeding, mimicking menses (92).

If the exposure to gonadotoxic treatments occurs in post-pubertal female patients, the potential clinical pictures encompasses either overt premature ovarian failure (POI, defined as the combination of oligo-amenorrhoea and raised FSH in the post-menopausal range in women <40 years), or a milder condition known as diminished ovarian reserve (DOR), a subclinical state defined as retained menses, normal FSH but reduced markers of ovarian reserve (i.e., low anti-Müllerian hormone and reduced antral follicular count on pelvis ultrasound). Women with DOR are at potential risk for impaired fertility and early detection of this condition could allow prompt undertaking of medically assisted fertility techniques by taking advantage of a potentially narrow window of opportunity that precedes the progression into POI and sterility.

According to various published analyses, the incidence of POI ranges from 44 to 100% among patients who received HSCT in childhood for either malignant or non-malignant haematological disorders (93–96). This wide range of variability is due to the clinical and demographicheterogeneity of different study cohorts. In female leukaemic patients, POI occurs in almost 100% of adolescents and young adults after TBI- or busulfan-based conditioning, while its incidence is remarkably lower after cyclophosphamide or melphalan (97–100). Preliminary data seem to show lower ovarian toxicity in female patients conditioned with treosulfan compared with busulfan (101).

Women with POI commonly experience clinical or sub-clinical signs and symptoms consistent with hypoestrogenism and need hormone replacement therapy, which is continued until the age when menopause is regarded as physiological.

Finally, female patients exposed to TBI experience a higher incidence of miscarriages, preterm deliveries, and obstetric complications, particularly during the third trimester of pregnancy, as a consequence of suboptimal uterine development. Conversely, no increased risk for malformations and genetic diseases in newborns is reported (102, 103).

#### Testicular Insufficiency

Alkylating agents and irradiation severely affect testicular function. Germ cells are remarkably more sensitive to chemotherapy and radiotherapy than testosterone-secreting Leydig cells; as a result, radiation doses as low as 2–6 Gy, busulfan >600 mg/kg and cyclophosphamide >7.5–9 g/m^2^ are the threshold above which spermatogonial cell depletion and subsequent oligo-azoospermia occurs (104, 105). Azoospermia has been reported in 85% of young male patients transplanted for either ALL, lymphoma or severe aplastic anaemia and exposed to TBI or abdominal irradiation before HSCT (106). Thus, semen collection and cryopreservation should always be recommended, when feasible, at diagnosis of malignancy.

Conversely, remarkably higher exposure is needed to affect testosterone secretion. In pre-pubertal boys, pubertal delay may occur at rates as high as 14% after cyclophosphamide alone, 48% after busulfan plus cyclophosphamide and 58% after fractionated 12–15.75 Gy TBI (91). Progressively increasing doses of testosterone are required to induce puberty and achieve secondary sexual features among those male peri-pubertal patients who present with overt pubertal arrest (91). The percentage of male patients requiring pharmacological induction of puberty is remarkably higher after 24 Gy testicular radiation for testicular relapse or disease (107). In a recent study conducted in 255 ALL survivors, overt testosterone deficiency was diagnosed in 71.4% of non-transplanted patients after 24-Gy testicular irradiation vs. 55.6% among TBI-conditioned and transplanted patients (108).

Among patients who have gone through puberty spontaneously, chemo conditioning may result in raised luteinizing hormone (a sign of subclinical damage) but testosterone secretion is generally retained (109). On the contrary, after TBI, with or without testicular radiation boost, adult men often experience a progressive decrease in testosterone levels, possibly associated to symptoms consistent with hypogonadism, thus indicating a need for life-long testosterone replacement therapy (110). As this may occur several years after exposure, life-long monitoring is required.

#### Impaired Glucose Metabolism and Dyslipidaemia

Hyperinsulinism, impaired glucose tolerance and diabetes present with a higher-than-expected incidence among transplanted ALL survivors, especially after TBI (111, 112). Early-onset hyperglycaemia during the early phase after allogeneic HSCT is commonly experienced as a result of treatment with immunosuppressive drugs, corticosteroids, parenteral nutrition and inflammatory cytokines associated with GvHD (113, 114).

In the long term, relative excess of adipose tissue is a well-known factor that predisposes to insulin resistance and diabetes (115). Although transplanted leukaemia and lymphoma survivors may present with normal body mass index, they can develop significant changes in their body composition, resulting in increased visceral fat and reduction of lean mass (116, 117). This condition, known as “sarcopenic obesity” leads to a relative decrease of myocyte insulin receptors vs. adipocyte receptors, which are remarkably less efficient at binding insulin. In addition, it has been demonstrated that when the pancreas falls within the irradiation field, as in TBI, β-cell reserve and overall pancreatic volume are remarkably lower than in controls, resulting in a higher incidence of pathological response to an oral glucose tolerance test (118).

Finally, low high-density lipoprotein and elevated triglyceride levels are reported in up to 30% of paediatric HSCT recipients, remarkably more frequently than in healthy controls (119).

Given the overall higher metabolic risk experienced after TBI-conditioned HSCT, the Children's Oncology Group recommend that healthcare professionals promote healthy lifestyle modifications to HSCT recipients and monitor patients' weight and body mass index annually and fasting blood glucose and glycated haemoglobin every 2 years (7).

### Eyes

Ocular complications after HSCT can affect all parts of the eye, from the cornea to the retina. Risk factors associated with ocular late effects are: specific conditioning regimens, immunosuppression, GvHD, and underlying disease (120, 121). Visual impairment can affect daily activities and, consequently, the QoL of patients.

#### Cataracts

Cataracts, characterised by an opacification of the lens, typically occur after TBI in >50% of cases, are related to radiotherapy dose, and increase over time. However, cataracts can also develop in children who have not received TBI in >25% of cases (122). Studies comparing cataract incidence between patients treated with single fraction TBI to those receiving fractionated TBI found a higher occurrence in the single-fraction groups (123). Horwitz et al. found that, with longer duration of follow-up in 201 children, cataract incidence after fractionated TBI (12 Gy in six fractions over 3 days) increased from 30% at 5 years post-transplant to 70.8% at 15 years (124). Long-term use of steroids is a cofactor for cataract formation, together with irradiation (23). Horwitz et al. could not detect a specific steroid dose effect (124). Another medication known to induce cataracts is busulfan, the chemotherapy-conditioning alternative to TBI, usually used in children younger than 4 years (24). The incidence is lower than after TBI, and severity of cataract is usually mild without needing surgery. Other causes of cataracts include high arterial blood pressure and metabolic syndrome, both of which often occur in HSCT recipients. The incidence of cataracts in children varies from 4 to 87%, depending on conditioning regimen and irradiation dose (125). The variations in range reported by different studies are due to the differences in conditioning regimens, radiation dose to the lens (eye shielding during TBI can be performed), differences in supportive care (such as use of steroids), and follow-up period as well as heterogeneity of study populations (126, 127). Van Kempen-Harteveld et al. demonstrated that 55% eye shielding reduced the incidence of cataracts from 90 to 31% in 188 paediatric patients who received single-fraction 8 Gy or two 6-Gy fractions of TBI (128). The risk of CNS relapse after eye shielding is negligible; in the leukaemia patients who received eye shielding, the incidence was 1.7% after 5 years (129).

#### Keratoconjunctivitis Sicca

Dry eye syndrome, also known as keratoconjunctivitis sicca, is the most frequent ocular complication after HSCT. It is characterised by insufficient tear production or excessive evaporation, with damage to the interpalpebral conjunctiva (3, 3, 130). Typical symptoms are itching, burning, a gritty feeling, or excessive tearing which sticks to lashes, photophobia, red eyes, impaired vision and pain (131). In children who have undergone HSCT, the reported incidence of keratoconjunctivitis sicca varies from 35 to 77% (126, 132–134). The most frequent cause of dry eyes is ocular GvHD, which typically develops 6–9 months after HSCT (135).

Incidence of ocular GvHD after HSCT amounts to 35% (132).

The National Institute for Health (NIH) criteria of 2015 define ocular GvHD as new onset, after HSCT, of dry, gritty or painful eyes, cicatricial conjunctivitis, keratoconjunctivitis, and confluent areas of punctate keratopathy. Schirmer's test is not recommended anymore for follow-up due to poor correlation with symptoms (131). Factors increasing the risk of ocular GvHD are the same risk factors for chronic GVHD overall, including, female donor transplanted to a male recipient, donor–recipient sex mismatch, increasing recipient age, higher numbers of CD34^+^ cells in the graft and peripheral blood stem cells as the graft source (132, 136–138). Recent studies showed that high number of CD3 cells in the graft were associated with a delay of lymphocytes recovery resulting in a higher incidence of acute GvHD Grad II or above (139). Routine eye examination before HSCT allows assessment of baseline conditions and annual ophthalmological screening after HSCT is recommended for early recognition of ocular complications after transplantation (131).

#### Microvascular Retinopathy

In 1983, ischemic microvascular retinopathy was described for the first time as a post-HSCT complication characterised by retinal cotton-wool patches, vitreous haemorrhage, and oedema of the optic disc (140). On ophthalmological examination, patients can be asymptomatic or complain of blurred vision or abnormalities in colour perception. Typically, ischaemic microvascular retinopathy occurs within 6 months of HSCT. The incidence ranges from 0 to 10% (141). Risk factors are use of TBI, cyclosporine A, busulfan, hypertension, diabetes, and hyperlipidiemia (121, 142, 143). The consequence is capillary damage in the ocular fundus. Symptoms can spontaneously regress, and a reduction of immunosuppression can lead to resolution of retinal lesions (144–146). Based on the potential risk factors it is important that calcineurin levels should be monitored, and that hypertension, diabetes or hyperlipidemia are treated.

#### Cytomegalovirus Uveitis/Retinitis

Cytomegalovirus retinitis (CMVR) is a rare sight-threatening manifestation after HSCT. Occurrence is typically late, with a mean time of 200 days post-transplant (147). Risk factors are young age at transplantation, pretransplant viremia, underlying primary immunodeficiency, non-myeloablative conditioning regimen, and acute GvHD (CD4 ≥ 200/ul) (147). Compared to patients with systemic infections, patients with CMVR had higher CD4 T-cell count (≥200/ul), expanding CD8 T- cell counts and lower CMV load. One hypothesis is that immunosuppression masks signs of inflammation (148). Most patients are asymptomatic for a long time, leading to a delayed diagnosis. The true incidence in children after HSCT is unknown due to the low number of published cases.

### Iron Overload

HSCT recipients often receive large red blood cell (RBC) transfusions both during the pre- and peri-transplant period. Accordingly, transfusion-related iron overload is listed among the commonest complications after HSCT, with a reported incidence ranging between 30 and 75% after allogeneic HSCT, with rates differing based on the diagnostic technique and criteria established (149–152).

A growing body of knowledge exists on the pathophysiology of iron-overload–induced tissutal toxicity: once transferrin is saturated, non-transferrin-bound iron becomes detectable, and—because of iron's ability to transfer electrons—this results in oxidative stress (153).

A potential worsening effect of infectious complications and GvHD on iron overload in the early post-HSCT period has been demonstrated. In addition, chemotherapy-induced mucositis may result in increased intestinal iron absorption. Finally, chemotherapy- and radiotherapy-associated hepatic damage may also contribute to the release of iron stores and diminish transferrin synthesis (154, 155).

It has been demonstrated that iron overload itself may play a contributory role on the pathogenesis of several early-onset complications of HSCT, such as invasive fungal infections (156), sepsis and sinusoidal obstruction syndrome (151, 157). However, assessing the interaction between pre- and post-transplantation ferritin levels and GvHD can be cumbersome, and no clear conclusions have been drawn to date about the potential detrimental effect of iron overload on GvHD.

Although advances in the supportive care and monitoring of long-term survivors have dramatically improved in the last decades, iron overload is still a challenging issue and may be associated with late sequelae such as liver fibrosis, hepatic focal nodular hyperplasia, heart failure, hypogonadism and diabetes (158, 159).

#### Diagnosis of Iron Overload

Theoretically, liver biopsy is the gold standard for evaluating iron tissue stores (152). Nevertheless, the need for a relatively large volume of tissue (4 mg wet weight), as well as the risks associated with this invasive procedure (with haemorrhage reported in about 0.5% of cases) make this diagnostic tool unappealing to most clinicians and patients (160).

Among the surrogate parameters developed to assess iron overload, serum ferritin is the most easily available and inexpensive. The European Society for Blood and Marrow Transplantation, Centre for International Blood and Marrow Transplant Research, and American Society for Transplantation and Cellular Therapy. 2006 guidelines for screening and prevention practises post HSCT promoted screening of serum ferritin levels to predict the risk of iron overload (32).

The ferritin level conventionally regarded as a threshold to prompt a complete assessment of iron overload is 1,000 ng/mL, although in patients with abnormal liver function tests, high transfusional needs or hepatitis C infection, this threshold should be lowered to 500 ng/mL (32, 159). Ferritin levels continue to be the mainstay for baseline clinical assessment of iron overload, although inflammation, ineffective erythropoiesis and liver disorders often result in raised ferritin levels (161–163). Accordingly, ferritin appears to show an overall unsatisfactory correlation with liver iron concentration (LIC) in paediatric patients, and LIC should always be assessed before undertaking any treatment for iron overload (150, 164).

T2^*^-weighted magnetic resonance imaging (MRI) has found a systematic clinical application in the last decade. LIC measurement by MRI has gained importance because it is non-invasive, rapid and widely available. Nowadays, T2 and R2 MRI techniques show a sensitivity and a specificity of 89 and 80% in the assessment of LIC, respectively. Ferritin levels of >1,000 ng/mL were found to correlate with a LIC of >7 mg/g in a population of patients transplanted for different haematological malignancies (165).

A superconducting quantum interference device (SQUID) can assess total body iron by biomagnetic susceptometric detection of the paramagnetic materials ferritin and hemosiderin. The iron content estimated shows a good correlation with LIC proven by biopsy. However, the SQUID technique has limitations: it is complex, expensive, and available in few centres worldwide (166). Busca et al. showed that LIC measurements obtained using a SQUID in the presence of moderate (LIC 1,000–2,000 μg Fe/g wet weight) or severe (LIC >2,000 μg Fe/g wet weight) iron overload were associated with high ferritin levels in 69% of patients (165).

#### Management of Iron Overload

Consensus about the indication and the best timing for treatment for iron overload after HSCT in ALL patients is lacking. Management of iron overload should be tailored based on several factors (i.e., the need for ongoing RBC transfusion therapy, ability to tolerate iron-depleting therapy, cost- effectiveness, and comorbidities). Phlebotomies and iron chelation agents are the two available treatment solutions. Experience-based recommendations suggest a combined aggressive approach in the case of severe iron overload with an estimated LIC >15 mg/g. When LIC is 7–15 mg/g dry weight, phlebotomy may be regarded as the best treatment solution. Among patients with milder iron overload (<7 mg/g), phlebotomies should be performed only in patients with concomitant liver disease (32, 159).

The safety and effectiveness of phlebotomies have been reported in adult survivors after HSCT, but only case series are available in paediatric patients (167–169). In patients who have achieved adequate engraftment and restored normal erythropoiesis following HSCT in paediatric age, phlebotomy represents a safe, and inexpensive approach. The need for an intravenous line and potentially poor compliance related to the high number of blood withdrawals required to achieve an effective depletion of iron storage represent the foremost limitations in childhood and adolescence, respectively (167).

Among chelators, deferoxamine is an iron-chelating agent available for intramuscular, subcutaneous or intravenous administration. Due to its short plasma half-life, deferoxamine should be administered at least 5 nights per week and be delivered by a subcutaneous pump for 8–12 h (170). This is the major restriction for wide administration in paediatric patients. A study assessing the efficacy of deferoxamine before and after HSCT in patients with thalassaemia showed that median serum ferritin 6 months after HSCT was statistically lower among treated patients (*p* = 0.007) than in the control group, without deferoxamine (170). Deferasirox is a tridentate iron chelator and has been licenced worldwide for the treatment of chronic iron overload in polytransfused patients aged ≥2 years. It is administered orally and the effective dose ranges between 20 and 40 mg/kg, with titration being guided by serum ferritin trends. Chelation with deferasirox after allogeneic HSCT was demonstrated to be effective and safe in reducing serum ferritin levels in two prospective, open-label, multicentre studies in adult patients who had received allogeneic HSCT and had iron overload (171, 172).

### Kidneys

Renal dysfunction is observed in up to 62% of transplanted cancer survivors and may be associated with a wide range of risk factors including nephrotoxic conditioning therapy for HSCT (high-dose chemotherapy and fractionated TBI), sinusoidal obstruction syndrome, hepatorenal syndrome, sepsis and corresponding antibiotic (aminoglycosides), and antifungal (amphotericin B) treatment (173). Some renal injury syndromes are probably related to cyclosporine A use, radiotherapy and GvHD (174, 175). It is also well-known that renal Fanconi syndrome may occur months or even years after the end of chemotherapy (176).

Renal disease after HSCT encompasses a wide spectrum of structural and functional abnormalities, ranging from vascular (hypertension, thrombotic microangiopathy) to glomerular (albuminuria, nephrotic glomerulopathies) to tubulo-interstitial lesions. All of these abnormalities may lead to a decreased glomerular filtration rate (GFR) and consequently chronic kidney disease (CKD). CKD defined by an elevated serum creatinine or a decreased GFR (<60 mL/min/1.73 m^2^) for ≥3 months develops in ~20% of long-term survivors following paediatric HSCT (177, 178). Three main clinical entities may be designated: thrombotic microangiopathy, nephrotic syndrome, and idiopathic CKD.

Thrombotic microangiopathy occurs in between 2 and 21% of HSCT recipients and represents a spectrum of clinical diseases characterised by systemic or intrarenal platelet aggregation, thrombocytopenia, and microvascular fragmentation of erythrocytes (179). Platelet aggregation can result in ischaemia and organ injury. When the presentation is fulminant, thrombotic microangiopathy is often associated with severe acute renal injury and death. The clinical course of thrombotic-microangiopathy-related kidney injury after HSCT is often an acute deterioration of renal function followed by a period of stabilisation and eventual development of CKD; full renal function is rarely restored (177).

The clinical manifestation of nephrotic syndrome includes proteinuria, oedema, hypoalbuminemia, and hypercholesterolaemia. The most common types of nephrotic syndrome that occur after HSCT are membranous nephropathy and minimal change disease. These are thought to be manifestations of GvHD in the kidney. Membranous nephropathy is characterised by the presence of immune complexes between the glomerular basement membrane and the podocytes, while minimal change disease is thought to be a T-cell–mediated process (180, 181).

There is a group of paediatric HSCT recipients who present with renal dysfunction not associated with thrombotic microangiopathy or nephrotic syndrome and, therefore, who are diagnosed with idiopathic CKD. The main risk factors predisposing HSCT recipients to idiopathic CKD are TBI, acute and chronic GvHD, and acute kidney injury (182).

As chronic renal impairment may occur in children who undergo HSCT with pre-transplant renal function within normal limits and regardless of conditioning regimen, screening of renal function (including blood pressure, renal function assessment, and if necessary kidney ultrasonography) is recommended in all paediatric HSCT recipients (183). In patients with renal insufficiency, nephrotoxic medication should be avoided; specific treatment strategies based on the specific diagnosis and its pathophysiology include immunosuppression for nephrotic syndrome, plasma exchange for thrombotic microangiopathy, and angiotensin-converting enzyme inhibitors or angiotensin receptor blockers for hypertension (177).

### Lungs

Pulmonary complications are among the most frequent serious sequelae after allogeneic HSCT for ALL. The two forms of chronic pulmonary dysfunction that are frequently observed are obstructive lung disease (OLD) and restrictive lung disease (RLD). The incidence of both forms ranges from 10 to 40% in all HSCT recipients and depends upon the donor source, the time interval after HSCT and presence of chronic GvHD (184). In both OLD and RLD, collagen deposition and the development of fibrosis (in the interstitial space in RLD or peri-bronchiolar space in OLD) are believed to contribute to the patterns of lung dysfunction displayed on pulmonary function tests (PFTs) (185, 186). Abnormalities in PFT parameters are not uncommon prior to HSCT and are generally thought to reflect exposure to infectious insults or previous chemotherapy.

Several studies have demonstrated OLD among recipients of HSCT during childhood (11, 187–189). Presence of chronic GvHD was the most consistent risk factor for development of OLD. The most common form of OLD after allogeneic HSCT is bronchiolitis obliterans (190). As mortality rates for bronchiolitis obliterans in paediatric HSCT patients range between 11 and 67%, all HSCT recipients should be carefully evaluated for this lung condition. Chest radiographs typically show hyperinflation, while mosaic perfusion is a common feature on high resolution computed tomography (CT), with decreased number and size of vessels causing parenchymal lucencies alongside normal lung tissue. Air trapping and bronchiectasis are also seen in bronchiolitis obliterans and may result in an air leak syndrome. Significant airway obstruction with bronchiolitis obliterans may be accompanied by only minimal radiographic findings. Serial PFT measurements may be more useful than imaging or histology for detecting the progression of bronchiolitis obliterans in children; however, due to technical difficulties in performing PFT in young children, the diagnosis of bronchiolitis obliterans is difficult and may be underreported (187).

RLD—as defined by a proportional decrease in forced vital capacity and forced expiratory volume in 1 second on spirometry and/or decreased total lung capacity using body plethysmography—has been described in many studies (11, 187, 188). Importantly, decline in total lung capacity or forced vital capacity occurring at 100 days and 1 year after HSCT is associated with an increase in non-relapse mortality (186). Patients who present with decreased pulmonary function may be at increased risk for sequelae from additional infectious or toxic exposures and should be counselled accordingly. Regular monitoring allows subtle changes in symptomatology or lung function to be detected (187). The most recognisable form of RLD is bronchiolitis obliterans organising pneumonia, characterised by dry cough, shortness of breath and fever. Radiographic findings show diffuse, peripheral, fluffy infiltrates consistent with airspace consolidation. Although reported in <10% of HSCT recipients, the development of bronchiolitis obliterans organising pneumonia is strongly associated with prior or ongoing acute or chronic GvHD (191).

Therapy for OLD defined by an irreversible airflow obstruction, as characterised by a forced expiratory volume in one second divided by forced vital capacity (FEV1/FVC) of <70%, and a FEV1 of <70% of predicted value, combines enhanced immunosuppression together with supportive care including antimicrobial prophylaxis, bronchodilator therapy and supplemental oxygen when indicated. While the approach to RLD is less well-defined, increasing evidence suggests that this form of pulmonary dysfunction may also be immunologically mediated. Unfortunately, the response to multiple immunosuppressive agents is limited and tends to occur only early in the course of treatment. The potential role for tumour necrosis factor α in the pathogenesis of both OLD and RLD suggests that neutralising agents such as etanercept may have promise (186, 191). Systematic post-transplant screening of lung function is essential for the diagnosis of early lung dysfunction and to facilitate optimal management. In cases of the development of respiratory symptoms or deterioration of lung function, rigorous clinical examination with an analysis of CT scan features and appropriate lung sampling should help the clinician make a specific diagnosis that considers long term pulmonary complications.

## Psychosocial Late Effects and Quality of Life After HSCT

The World Health Organisation defines QoL as “an individual's perception of their position in life in the context of the culture and value systems in which they live and in relation to their goals, expectations, standards and concerns.” More specifically, health-related QoL is defined as “the extent to which usual or expected physical, emotional, and social well-being are affected by a medical condition or its treatment.” The concept of health-related QoL encompasses physical, cognitive, emotional, and social functioning and well-being; it has emerged as a significant area of research that now is recognised as an important endpoint for many studies alongside survival endpoints (192).

Factors adversely affecting QoL and social challenges in transplanted survivors are amenable to intervention. Therefore, there is a need to incorporate effective interventions in the routine follow-up care of paediatric allogeneic HSCT recipients in order to improve their QoL and enhance their psychological and interpersonal growth. Psychosocial late effects include post-traumatic stress symptoms, low self-esteem, and lower QoL. Social problems also have been documented among survivors in terms of social anxiety, poor peer acceptance and self-perception issues. Children transplanted due to ALL are at especially high risk due to their average older age at the time of diagnosis, type and length of pretransplant treatment, severity of disease, length of remission or shorter time since diagnosis, and the medical late effects of disease and treatment (193).

Studies focused on QoL and psychosocial sequelae in paediatric ALL patients undergoing HSCT establish these endpoints as relevant fields of enquiry. Increasing attention in these fields derives from the success of curative ALL therapies, including HSCT, in recent years. Following HST, survivors can gradually restart their normal activities, with the consequence that psychological aspects linked to therapy must now be considered for these patients alongside medical late effects, in order to better understand how patients might adapt and be supported. The most traumatic period from a psychological point of view is often the period in which children and parents spend isolated in the transplant unit in the early post-transplant period. Longitudinal, prospective studies of variations in QoL along the various steps of HSCT and long-term follow-up may allow us to conceptualise how psychosocial aspects of patients' lives and development are affected over time (20, 194, 195).

## Transition From Paediatric to Adult HSCT Care

Given the prevalence and impact of complications following HSCT during childhood it is now widely recognised that survivors must have timely access to life-long care in order to prevent, ameliorate and manage the adverse late effects of transplant. In the same way that it is accepted that children are optimally treated by healthcare providers trained and experienced in the care of children, so too, adults should be cared for by those trained and experienced in adult medicine (196). Therefore, as more and more children survive, the transition of adolescents from paediatric to adult services is crucial.

This healthcare transition comes at a time when adolescents are already facing the challenges of transitioning from childhood to adulthood (197). The adolescent brain is different to the brain in childhood or adulthood contributing to the vulnerability of adolescents to risk taking and poor self-regulation (198). While for healthy adolescents, risk taking and poor self-regulation might involve social behaviours such as alcohol and illicit drug taking, unprotected sex and reckless driving, for those with chronic medical conditions the fallout can include a lack of compliance with healthcare. For these reasons, it has long been recognised that the transition of healthcare for adolescents and young adults with chronic healthcare conditions needs to planned, purposeful and well-supported in order for it to be successful (196, 199–204).

The American Academy of Paediatrics, American Academy of Family Physicians and American College of Physicians published a consensus statement in 2002 on healthcare transition for young adults with special healthcare needs (196). This statement recommends some critical initial steps to ensure uninterrupted, developmentally appropriate care as patients move from adolescence to adulthood. These steps include: identification of appropriate healthcare providers in the adult system, developmentally appropriate education of the adolescent/young adult, a written healthcare transition plan and in countries without universal health care, access to adequate healthcare insurance.

Unlike most adolescent/young adults with chronic medical conditions, paediatric HSCT recipients, for the most part, do not have acute healthcare needs. Rather—similar to other childhood cancer survivors—they are a unique group of patients that require significant preventative healthcare. Their healthcare requirements focus on surveillance, prevention and education, rather than just treatment.

There is little in the literature looking specifically at transition of paediatric HSCT recipients to adult services. There is more in the literature for other childhood cancer survivors, who are a larger but similar population. A recently published review looked at the transition of childhood cancer survivors to adult healthcare (205). The authors looked at 26 studies focused on three main areas: transition practises, transition readiness tools and barriers to successful transition. There were three main models of transition: (1) a direct transition from paediatric to adult oncology units; (2) transfer to care under primary care physicians, with referral to adult medical specialists as needed; and (3) shared care, where the primary care physician works in collaboration with an oncology unit. Transition tools are an important part of planning and supporting successful transition (206–211). The tools, which included workbooks, questionnaires and scales, aimed to assess the readiness of the childhood cancer survivor to transition. The tools are useful in identifying areas in which an individual survivor needs more support or education. The most frequently identified barriers to transition in these publications related to knowledge, education and empowerment of the survivors as well as the knowledge and education of healthcare providers (193, 194, 212–223). This highlights that education of both survivors and their healthcare providers is an integral part of successful transition.

Importantly, there is nothing to our knowledge in the literature evaluating the success or failure of transition processes and methods to manage those that are lost to follow-up.

In our review of the literature we identified two publications addressing transition specifically in survivors of HSCT in childhood. Hashmi et al. (20) looked at the need for long-term follow-up after HSCT in adults and childhood and the need for transition of care from overworked and under-resourced HSCT units. They highlighted that vulnerable transitions included paediatric patients transitioning to adulthood, noting the lack of clear transition pathways. This group discussed the importance of written survivorship care plans that are individualised for each patient. Written individualised care plans are one of the integral steps recommended in the 2002 consensus statement on transition of children with long-term special healthcare needs to adult services (196). The North American Children's Oncology Group guidelines on the long-term follow-up of survivors of childhood, adolescent and young adult cancers (www.survivorshipguidelines.org) include instructions to develop individualised written care recommendations (196).

Cupit et al. reviewed the long-term healthcare needs of childhood bone and marrow transplantation survivors and also touched on the issues of transition, preventative healthcare and access to health insurance (192). They tailored the steps recommended in the 2002 consensus statement (196) to issues specific to transition of HSCT recipients. This provides a good framework for transition for this patient population. The steps are as follows: 1. Identification of a health care provider who will assume responsibility for current and future health care, 2. Individualised care plan which outlines the therapy received, any complications experienced and recommend surveillance for potential long-term complications, 3. Health care transition plan which should be written well prior to transition and should be discussed with the patient and their family. The responsibilities of the various health care providers should be made clear in this document. Both the care plan and transition plans should be updated regularly if there are any significant changes and these changes communicated with the patient. 4. Ensuring access to adequate health care insurance. This step is relevant to patient in countries without universal health care. 5. Communication. This is perhaps the most important, clear and well-documented communication with the patient, their family and the adult health care providers is imperative for smooth and successful transition.

In summary, clear transition pathways from paediatric to adult healthcare for survivors who have undergone HSCT during childhood is necessary to ensure healthcare continuity, avoid preventable poor outcomes and promote early identification and management of long-term complications. However, transition is only successful where it is planned, anticipated, purposeful and follows clear pathways and where the services that patients transition to and from are adequately resourced. Education, which has been found to be a critical successful factor of transition, is paramount. This includes education of not just the survivors but also the healthcare providers who will be looking after them. A written individualised survivorship care plan is an important resource in that education process.

## Conclusion

Transplanted ALL survivors are a growing vulnerable population worldwide. They are at risk of long-term sequelae that can often appear years or even decades after HSCT and that can impact on quality of life. As such, survivors require life-long risk-adapted follow-up. Alertness and early detection of late effects allows management to mitigate some long-term consequences of ALL and HSCT.

Regular exchange with patients and their families enables healthcare providers to follow patients throughout their life after a relevant event like HSCT, train them on a healthy lifestyle practises and teach them how to take over responsibilities towards themself for periodical controls and potential health issues. All survivors should receive a summary of the treatment they have received and a risk-adapted care plan. This individualised follow-up proposal should be explained to each individual for better understanding of its possible implications. Moreover, interdisciplinary transition consultation is recommended for smooth transfer to adult care.

Long-term outcomes should be documented in multicentre prospective trials to understand better pathophysiological pathways and predisposing factors for late effects and to optimise the future management of HSCT recipients.

## Author Contributions

All authors listed have made a substantial, direct, and intellectual contribution to the work and approved it for publication.

## Conflict of Interest

The authors declare that the research was conducted in the absence of any commercial or financial relationships that could be construed as a potential conflict of interest. The reviewer GL declared a past collaboration with one of the authors TD-F to the handling Editor.

## Publisher's Note

All claims expressed in this article are solely those of the authors and do not necessarily represent those of their affiliated organizations, or those of the publisher, the editors and the reviewers. Any product that may be evaluated in this article, or claim that may be made by its manufacturer, is not guaranteed or endorsed by the publisher.
